# Mitochondrial CB1 receptor is involved in ACEA-induced protective effects on neurons and mitochondrial functions

**DOI:** 10.1038/srep12440

**Published:** 2015-07-28

**Authors:** Lei Ma, Ji Jia, Wen Niu, Tao Jiang, Qian Zhai, Lei Yang, Fuhai Bai, Qiang Wang, Lize Xiong

**Affiliations:** 1 Department of Anesthesiology, Xijing Hospital, The Fourth Military Medical University, Xi’an, Shaanxi, China, 710032; 2 Department of Pathology and Pathophysiology, The Fourth Military Medical University, Xi’an, Shaanxi, China, 710032

## Abstract

Mitochondrial dysfunction contributes to cell death after cerebral ischemia/reperfusion (I/R) injury. Cannabinoid CB1 receptor is expressed in neuronal mitochondrial membranes (mtCB1R) and involved in regulating mitochondrial functions under physiological conditions. However, whether mtCB1R affords neuroprotection against I/R injury remains unknown. We used mouse models of cerebral I/R, primary cultured hippocampal neurons exposed to oxygen-glucose deprivation/reoxygenation (OGD/R) and Ca^2+^-induced injury in purified neuronal mitochondria to investigate the role of mtCB1R in neuroprotection. Our results showed selective cell-permeant CB1 receptor agonist, arachidonyl-2-chloroethylamide (ACEA), significantly up-regulated the expression of mtCB1R protein in hippocampal neurons and tissue. *In vitro*, ACEA restored cell viability, inhibited generation of reactive oxygen species (ROS), decreased lactate dehydrogenase (LDH) release and reduced apoptosis, improved mitochondrial function. *In vivo*, ACEA ameliorated neurological scores, diminished the number of TUNEL-positive neurons and decreased the expression of cleaved caspase-3. However, ACEA-induced benefits were blocked by the selective cell-permeant CB1 receptor antagonist AM251, but just partially by the selective cell-impermeant CB1 receptor antagonist hemopressin. In purified neuronal mitochondria, mtCB1R activation attenuated Ca^2+^-induced mitochondrial injury. In conclusion, mtCB1R is involved in ACEA-induced protective effects on neurons and mitochondrial functions, suggesting mtCB1R may be a potential novel target for the treatment of brain ischemic injury.

Cardiac arrest-induced neurologic injury is a vital cause of death among the patients with a successful cardiopulmonary resuscitation^1^. American Heart Association and related organizations have made long-term efforts to improve resuscitation guidelines; however, the hospital mortality remains proximate 70% in patients resuscitated successfully^2^. Unfortunately, about 2/3 of the survivors may have moderate to severe cognitive deficits^3^. Therefore, searching for effective therapeutics which can alleviate neurologic injury induced by cerebral I/R is of great importance.

Mitochondria are cellular energy factories, participating in oxidative stress, calcium homeostasis, generation of reactive oxygen species (ROS) and programmed cell death, which are crucial in regulating brain functions^4^,^5^. In cerebral I/R injury, mitochondrial dysfunction leads to neuronal death^6^. More importantly, several studies showed that mitochondrial damage occurs within 4–6 hours after the onset of cerebral ischemia, which provides a promising strategy for targeting mitochondria to develop cerebral I/R injury therapeutics^7^,^8^,^9^. However, approaches that regulate mitochondria effectively after cerebral I/R injury are still poorly understood.

Cannabinoid CB1 receptor is a G protein-coupled receptor expressed mainly in neuronal plasma membrane, modulating neuronal metabolism, activity and functions strictly^10^,^11^,^12^. Early studies indicated that the marijuana-derivative cannabis Δ9-tetrahydrocannabinol (THC) could induce neuroprotection by up-regulating CB1 receptor and maintain mitochondrial function^10^,^13^,^14^. In addition, recent evidence also showed that CB1 receptor can adjust mitochondrial biogenesis in non-neural tissues^15^,^16^. However, THC may be of limited use due to its side effects, including addiction, psychoactivity, tolerance and cytotoxicity^17^,^18^. In 2012, Marsicano *et al.* found that CB1 receptor is also expressed in neuronal mitochondrial membranes (mtCB1R), and up-regulation of which regulates mitochondrial respiration and energy metabolism under physiological conditions^19^. However, whether up-regulation of mtCB1R modulates mitochondrial functions and exerts neuroprotection against I/R injury is still unknown.

In the present study, we used mouse models of global cerebral I/R, primary cultured hippocampal neurons OGD/R and Ca^2+^-induced injury in purified intact mitochondria from hippocampal neurons to investigate the role of mtCB1R in neuroprotection and improvement of mitochondrial function.

## Results

### ACEA-induced up-regulation of mtCB1R was blocked by AM251, but not by hemopressin

**Figure 1:**
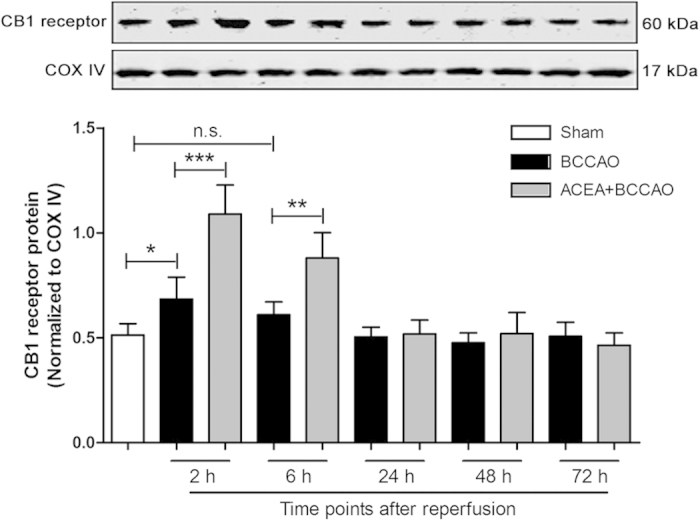
Expression of mtCB1R protein in mouse hippocampus at different time points after reperfusion. Western blot showing mtCB1R protein expression at 2 h, 6 h, 24 h, 48 h and 72 h after reperfusion respectively. Data represent mean ± SD. *P < 0.05; **P < 0.01; ***P < 0.001; n.s.: no significance. COX IV: cytochrome c oxidase; BCCAO: bilateral common carotid artery occlusion.

First, we attempted to detect the expression of mtCB1R at different time points after cerebral I/R. Mitochondria protein were isolated at 2 h, 6 h, 24 h, 48 h and 72 h after reperfusion respectively (Fig. 1). We observed that mtCB1R expression was significantly increased at 2 h after reperfusion compared with Sham group (*P* = 0.046) and ACEA increased the mtCB1R expression more obviously (*P* < 0.001). ACEA also up-regulated the mtCB1R expression at 6 h after reperfusion compared with Sham group (*P* < 0.001). However, there was no significant difference in mtCB1R protein expression at the other time points.

**Figure 2:**
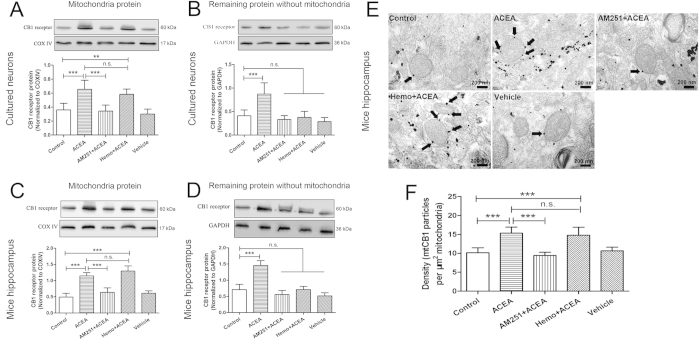
Expression of CB1 receptor protein in primary cultured hippocampal neurons and mouse hippocampus. (**A**, **C**) Western blot showing mtCB1R protein expression at 2 h after administration *in vitro and in vivo* (n = 5). (**B**, **D**) Western blot showing CB1 protein expression in the remaining samples without mitochondria protein at 2 h after administration *in vitro and in vivo* (n = 5). **(E)** Immunogold electron microscopy showing mtCB1R protein expression. **(F)** Density of mtCB1R immunoparticles were calculated per area of mitochondria (n = 3). Scale bars = 200 nm. Data represent mean ± SD. **P < 0.01; ***P < 0.001; n.s.: no significance. COX IV: cytochrome c oxidase; Hemo: hemopressin.

Subsequently, we investigated the effect of ACEA on mtCB1R expression. Previous studies have reported that cannabinoid could increase the expression of CB1 receptor protein and the increased CB1 receptor was functional^20^,^21^. In the present study, we isolated mitochondria protein at 2 h after ACEA administration to detect the expression of mtCB1R. As shown in Fig. 2A,C, mtCB1R expression was significantly increased at 2 h after ACEA treatment compared with Control group in hippocampal neurons and mouse hippocampus (*P* < 0.001). The effects were reversed by the cell-permeant CB1 receptor antagonist, AM251 (*P* < 0.001), but not by the cell-impermeant CB1 receptor antagonist, hemopressin (*P* = 0.24 and *P* = 0.105). There were no differences in mtCB1R expression between Control group and Vehicle group (*P* = 0.333 and *P* = 0.18). In the remaining samples without mitochondria protein, CB1 receptor expression increased just in ACEA group (Fig. 2B,D, *P* < 0.001), but not in AM251 + ACEA, Hemo + ACEA or Vehicle groups, compared with Control group (*P* = 0.449 and 0.125, *P* = 0.735 and 0.979, *P* = 0.237 and 0.054, respectively).

CB1 receptor immunoreactivity was detected by immunogold electron microscopy in the mouse CA1 hippocampal neurons (Fig. 2E). We observed intracellular CB1 receptor was expressed on the mitochondrial membrane, and the density of mtCB1R was significantly increased in ACEA and Hemo + ACEA groups compared with Control group (Fig. 2F, *P* < 0.001), but not in AM251 + ACEA and Vehicle groups (*P* = 0.424 and *P* = 0.603).

**Figure 3:**
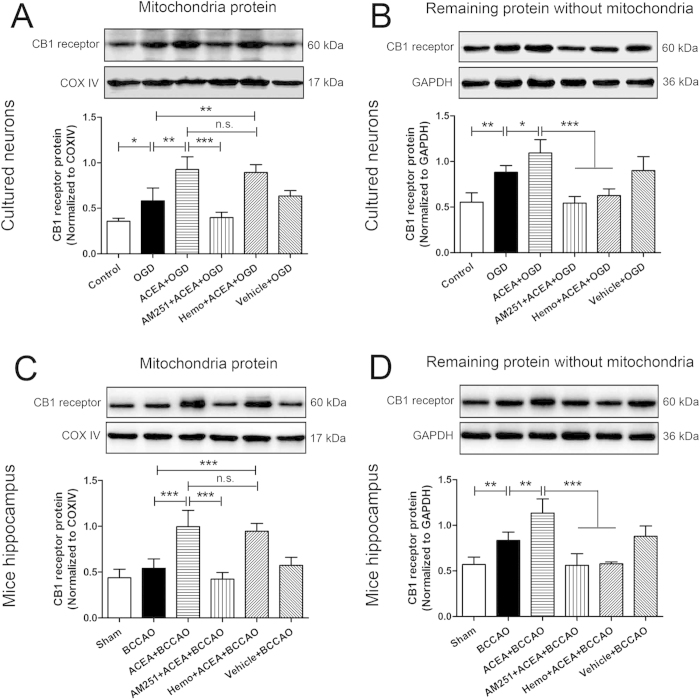
Expression of CB1 receptor protein in primary cultured hippocampal neurons at 2 h after reoxygenation and mouse hippocampus at 2 h after reperfusion. (**A**, **C**) Western blot showing mtCB1R protein expression *in vitro and in vivo* (n = 5). (**B**, **D**) Western blot showing CB1 protein expression in the remaining samples without mitochondria protein *in vitro and in vivo* (n = 5). Data represent mean ± SD. *P < 0.05; **P < 0.01; ***P < 0.001; n.s.: no significance. COX IV: cytochrome c oxidase; OGD: oxygen-glucose deprivation; Hemo: hemopressin; BCCAO: bilateral common carotid artery occlusion.

To detect the CB1 receptor expression after OGD/R or I/R, mitochondria were isolated at 2 h after reoxygenation or reperfusion to detect the expression of mtCB1R. As shown in Fig. 3A,C, mtCB1R expression was significantly increased in OGD group compared with Control group (*P* = 0.01). ACEA increased the mtCB1R expression further compared with OGD and BCCAO groups respectively (*P* = 0.002 and *P* < 0.001). The effects were abolished by AM251 (*P* < 0.001), but not by hemopressin (*P* = 0.575 and *P* = 0.47). In the remaining samples without mitochondria protein, CB1 receptor expression was also increased at 2 h after reoxygenation or reperfusion (Fig. 3B,D, *P* = 0.001 and *P* = 0.003), and ACEA increased the expression further (*P* = 0.014 and *P* = 0.001). While treatment with AM251 or hemopressin abolished the effects of ACEA (*P* < 0.001).

### ACEA-induced cytoprotection against OGD/R injury was reversed by AM251, but just partially by hemopressin

**Figure 4:**
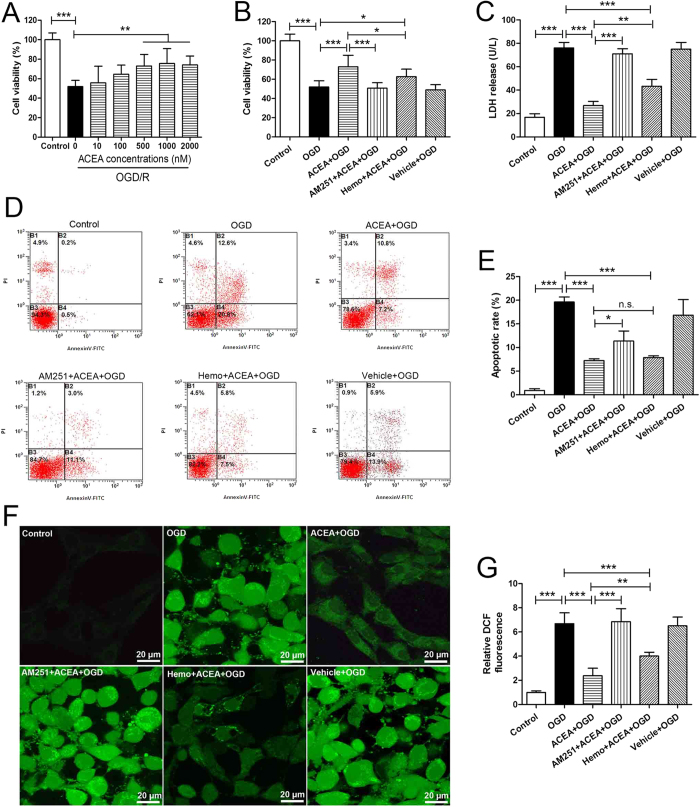
ACEA-induced cytoprotection against OGD/R injury in primary cultured hippocampal neurons. (**A**) The effective dose of ACEA against OGD/R evaluated by WST-8 (n = 8). Cell injury was evaluated in terms of WST-8 cell viability (**B**), (n = 8), LDH release (**C**), (n = 6), apoptosis rate (**D**, **E**), (n = 5) and intracellular ROS level (**F**, **G**), (n = 6) at 24 h after reoxygenation. Scale bars  = 20 μm. Data represent mean ± SD. *P < 0.05; **P < 0.01; ***P < 0.001; n.s.: no significance. OGD/R: oxygen-glucose deprivation/reoxygenation; WST-8: 2-(2-methoxy-4-nitrophen​yl)-3-(4-nitrophenyl)-5-(​2,4-disulfophenyl)-2H-tet​razolium, monosodium salt; Hemo: hemopressin; LDH: lactate dehydrogenase; ROS: reactive oxygen species.

Next, we asked whether up-regulation of mtCB1R could induce neuroprotection *in vitro*. To find a suitable ACEA concentration, hippocampal neurons were exposed to ACEA at 10 nM, 100 nM, 0.5 μM, 1 μM and 2 μM at the onset of reoxygenation. Cell viability was significantly increased in the presence of ACEA at 0.5 μM, 1 μM and 2 μM (Fig. 4A, *P* = 0.003, 0.001 and 0.002) and the dose of 1 μM was used in the subsequent experiments.

The injury of neurons was assessed at 24 h after reoxygenation. The apoptotic rate of neurons was analyzed by flow cytometry and the lower right quadrant represented the percentage of early apoptotic cells. ACEA increased cell viability, attenuated LDH release, decreased apoptotic rate and inhibited generation of intracellular ROS in the cells exposed to OGD/R (Fig. 4B–G, *P* < 0.001). The protective effects of ACEA were abolished by the cell-permeant CB1 receptor antagonist, AM251 (*P* < 0.001, *P* < 0.001, *P* = 0.011 and *P* < 0.001), while not or just partially reversed by the cell-impermeant CB1 receptor antagonist, hemopressin. There were no statistical differences in the cell viability, LDH release, apoptotic rate or intracellular ROS between OGD and Vehicle + OGD groups (*P* = 0.502, *P* = 0.782, *P* = 0.063 and *P* = 0.892, respectively).

### ACEA-induced neuroprotection against cerebral I/R injury was reversed by AM251, but just partially by hemopressin

**Figure 5:**
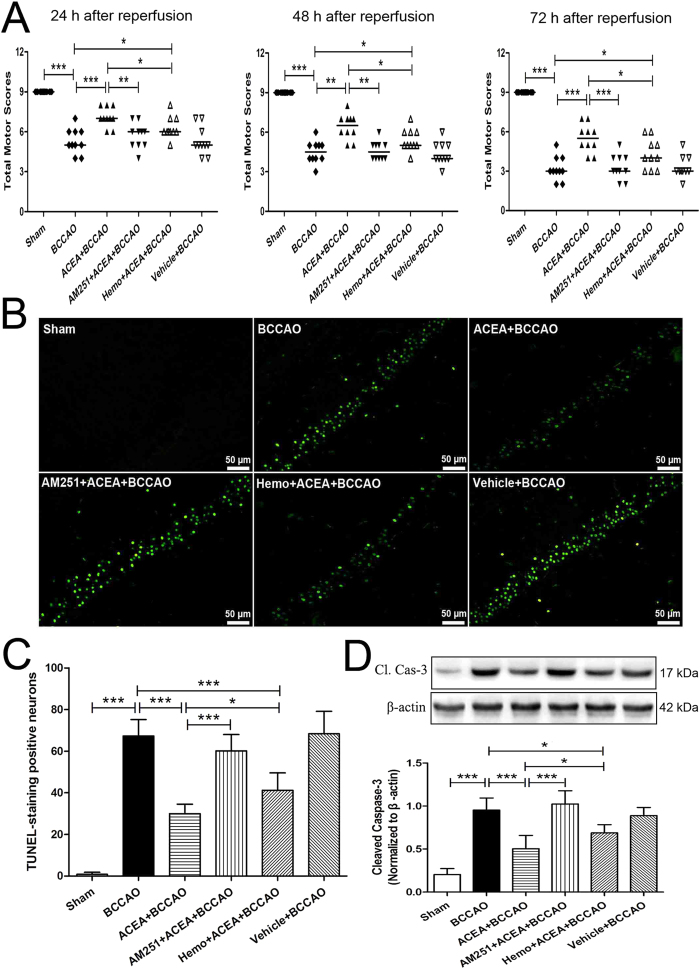
ACEA-induced neuroprotection against cerebral ischemia/reperfusion injury in mice. (**A**) Neurological assessment at 24 h, 48 h and 72 h after reperfusion (n = 13). Data represent the median. (**B**) Representative photomicrographs of TUNEL staining in hippocampal CA1 region. Scale bars = 50 μm. (**C**) Quantitative analysis of the number of TUNEL-positive cells in hippocampal CA1 region at 72 h after reperfusion (n = 5). (**D**) Western blot showing representative the expression of cleaved caspase-3 (Cl. Cas-3) protein in the hippocampus at 72 h after reperfusion (n = 5). Data represent mean ± SD. *P < 0.05; **P < 0.01; ***P < 0.001. BCCAO: bilateral common carotid artery occlusion; Hemo: hemopressin.

Subsequently, we investigated the effect of mtCB1R up-regulation on neuroprotection in mice. All animals survived until the final neurological assessment at 72 h after reperfusion. The neurological scores in ACEA + BCCAO group were significantly higher than those of BCCAO group at 24, 48, and 72 h after reperfusion respectively (Fig. 5A, *P* < 0.001, *P* = 0.002 and *P* < 0.001). The cell-permeant CB1 receptor antagonist AM251 reversed the ACEA-induced benefits at the three time-points (*P* = 0.004, *P* = 0.001 and *P* < 0.001), while the cell-impermeant CB1 receptor antagonist hemopressin just partially reversed the benefits. No significant differences in the neurological scores were observed between BCCAO and Vehicle + BCCAO groups at the three time-points (*P* = 0.912, *P* = 0.796, *P* = 0.766, respectively).

The number of TUNEL-positive neurons in the hippocampal CA1 region (Fig. 5B,C) and the expression of cleaved caspase-3 in the hippocampus (Fig. 5D) were significantly increased at 72 h after reperfusion in BCCAO group compared with that of Sham group (*P* < 0.001). ACEA diminished the number of TUNEL-positive neurons and decreased the expression of cleaved caspase-3 induced by BCCAO (*P* < 0.001). The benefits of ACEA were blocked by the cell-permeant CB1 receptor antagonist, AM251 (*P* < 0.001), but just partially by the cell-impermeant CB1 receptor antagonist, hemopressin. No significant differences were found in the number of TUNEL-positive neurons or the cleaved caspase-3 expression between BCCAO and Vehicle + BCCAO groups (*P* = 0.818, *P* = 0.461, respectively).

### ACEA-induced improvement of mitochondrial function was blocked by AM251, but just partially by hemopressin

In order to determine whether ACEA-induced neuroprotection was accompanied by the improvement of mitochondrial function, we observed the mitochondrial ultrastructure in mouse CA1 hippocampal neurons, measured the activities of complexes I, II, and IV of mitochondrial electron transport chain and the mitochondrial membrane potential (MMP) in purified mitochondria from primary cultured hippocampal neurons.

**Figure 6:**
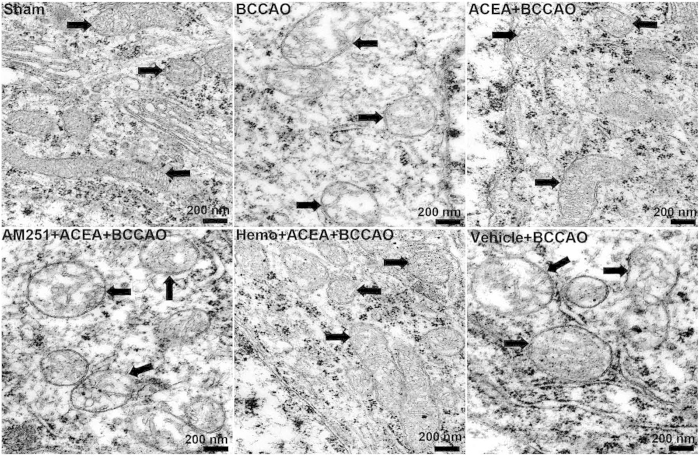
Transmission electron micrographs showing mitochondrial ultrastructure. Representative electron microphotographs show mitochondrial ultrastructure in mouse CA1 hippocampal neurons at 72 h after the reperfusion (n = 3). Mitochondrial ultrastructures are indicated by arrows. Scale bars = 200 nm. BCCAO: bilateral common carotid artery occlusion; Hemo: hemopressin.

The mitochondria in Sham group were elongated or round and had numerous cristae with parallel alignment. The outer and inner membranes of mitochondria were clearly distinguishable. Mitochondria from BCCAO, AM251 + ACEA + BCCAO and Vehicle + BCCAO groups showed swelling, vacuolization, disruption and loss of cristae. In ACEA + BCCAO and Hemo + ACEA + BCCAO groups, the mitochondrial structure was almost normal, but the cristae were slightly disrupted (Fig. 6).

**Figure 7:**
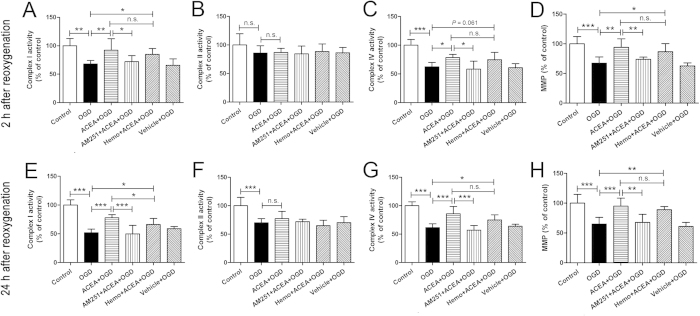
Effects of ACEA on mitochondrial function at 2 and 24 h after reoxygenation. Neuronal mitochondria were purified at 2 and 24 h after reoxygenation respectively, and the activities of complexes I (**A**, **E**), II (**B**, **F**) and IV (**C**, **G**) were measured spectrophotometrically; the mitochondrial membrane potential (MMP) (**D**, **H**) was evaluated by fluorescence spectrophotometry. Data represent mean ± SD (n = 6). *P < 0.05; **P < 0.01; ***P < 0.001; n.s.: no significance. OGD: oxygen-glucose deprivation; Hemo: hemopressin.

At 2 and 24 h after reoxygenation (Fig. 7), the activities of complexes I and IV and the MMP showed significant reduction in OGD group, compared with Control group (2 h: *P* = 0.001, *P* < 0.001 and *P* < 0.001; 24 h: *P* < 0.001). And ACEA improved the mitochondrial functions significantly (2 h: *P* = 0.006, *P* = 0.017 and *P* = 0.001; 24 h: *P* < 0.001). The benefits of ACEA were completely reversed by the cell-permeant CB1 receptor antagonist, AM251 (2 h: *P* = 0.019, *P* = 0.012 and *P* = 0.006; 24 h: *P* < 0.001, *P* < 0.001 and *P* = 0.001), while not or just partially by the cell-impermeant CB1 receptor antagonist, hemopressin. No significant differences were observed between OGD and Vehicle + OGD groups (2 h: *P* = 0.788, *P* = 0.787 and *P* = 0.499; 24 h: *P* = 0.225, *P* = 0.652 and *P* = 0.565). The OGD-induced decrease in complex II activity was observed at 24 h after reoxygenation (Fig. 7F, *P* < 0.001), but not at 2 h (Fig. 7B, *P* = 0.102); however, ACEA did not alleviate the decrease of complex II activity (*P* = 0.288).

### ACEA attenuates Ca^2+^-induced mitochondrial dysfunction in purified intact mitochondria from hippocampal neurons

**Figure 8:**
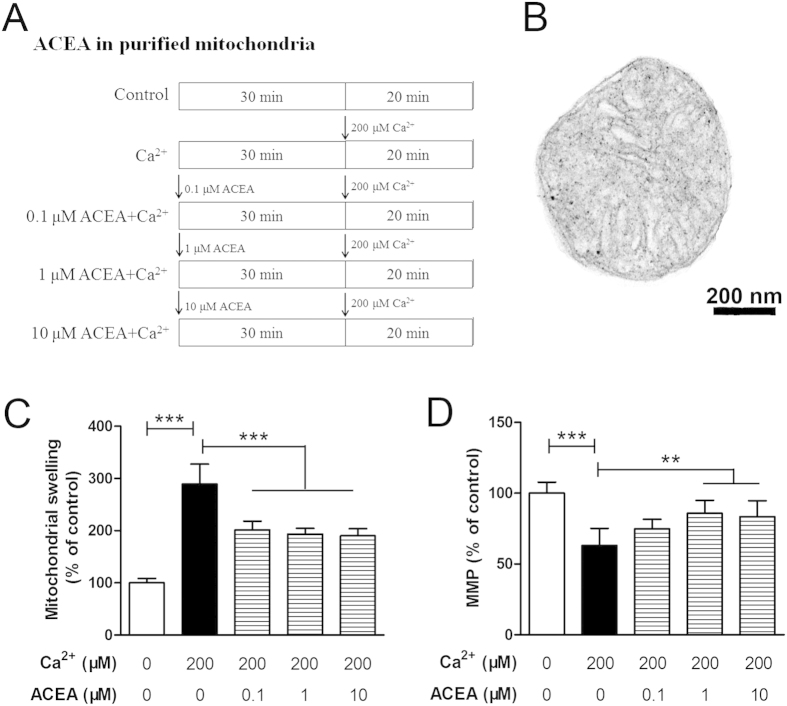
Effects of ACEA on Ca^2 + ^-induced mitochondrial injury *in vitro*. (**A**) Protocol. (**B**) Representative electron micrograph of purified intact mitochondria from normal hippocampal neurons. Scale bars = 200 nm. Mitochondrial swelling was measured spectrophotometrically (**C**) and mitochondrial membrane potential (MMP) was evaluated by a fluorescence spectrophotometer (**D**). Data represent mean ± SD (n = 6). **P < 0.01; ***P < 0.001.

To further clarify the protection of mtCB1R activation against mitochondrial injury, intact mitochondria were obtained from normal hippocampal neurons to investigate the direct protective effects of ACEA-induced mtCB1R activation on mitochondrial functions^19^. Purified mitochondria were treated with a high concentration of Ca^2+^ to mimic the disorder of Ca^2+^ homeostasis, which is thought to be an important inducer in cerebral I/R injury^22^,^23^. Mitochondrial suspensions were divided into five groups (n = 6, Fig. 8A) and the purified mitochondria were round or oval with the morphological integrity of membrane and cristae structures (Fig. 8B). Except for the Control and the Ca^2+^ only groups, the other three groups of mitochondrial suspensions received 0.1 μM, 1 μM and 10 μM ACEA, respectively, at 30 min before Ca^2 + ^administration. Ca^2+^ induced a noticeable mitochondrial swelling, as evidenced by the reduction in absorbance at 520 nm (Fig. 8C, *P* < 0.001), which was followed by a decline of MMP (Fig. 8D, *P* < 0.001). ACEA markedly prevented Ca^2+^-induced mitochondrial swelling (*P* < 0.001) and increased the MMP at the doses of 1 and 10 μM (P = 0.002 and 0.004).

## Discussion

In the present study, we found that mtCB1R expression was significantly up-regulated after the administration of ACEA, leading to neuroprotection against OGD/R and cerebral I/R injury. These benefits of ACEA were abolished by the selective cell-permeant CB1 receptor antagonist, AM251, but not or just partially by the selective cell-impermeant CB1 receptor antagonist, hemopressin. Furthermore, in purified intact mitochondria from normal hippocampal neurons, ACEA-induced mtCB1R activation markedly prevented Ca^2+^-induced mitochondrial swelling and increased the membrane potential. These findings suggest that up-regulation of mtCB1R is involved in ACEA-induced protective effects on neurons and mitochondrial functions, indicating mtCB1R could be a potential novel target for the treatment of brain ischemic injury.

Early studies presumed that CB1 receptor is mainly expressed in neuronal plasma membrane and CB1 receptor activation can induce neuroprotection against ischemic brain injury^24^,^25^. A recent research showed that CB1 receptor is also present in neuronal mitochondrial membrane, where the receptor directly regulates mitochondrial respiration and energy metabolism under physiological conditions^19^. However, whether the neuroprotection is mediated by mtCB1R is still not clear. After the administration of cell-permeant CB1 receptor agonist ACEA, a high-selective CB1 receptor agonist^26^,^27^, the expression of mtCB1R protein was significantly increased in hippocampal neurons and mouse hippocampus. By using immunogold electron microscopy, we found that ACEA obviously up-regulated mtCB1R expression on the mitochondrial membrane of mouse CA1 hippocampal neurons. Previous studies have showed that ACEA increased the expression of CB1 receptor protein and the increased CB1 receptor was functional^20^,^21^. Thus, it is generally considered that activation of CB1 receptor is positive correlation with the up-regulation of CB1 receptor expression. Moreover, we found that ACEA-induced mtCB1R protein up-regulation was reversed by the selective cell-permeant CB1 receptor antagonist AM251^27^,^28^, but not by the selective cell-impermeant CB1 receptor antagonist hemopressin^29^, suggesting mtCB1R may be up-regulated selectively.

We then investigated whether increased expression of mtCB1R can induce neuroprotection. C57BL/6 mouse has been reported to be highly prone to neuronal damage after cerebral ischemia because of the poorly developed anastomosis between the carotid artery (posterior communicating artery)^30^,^31^ and the bilateral common carotid artery occlusion (BCCAO) in C57BL/6 mouse was used widely as a model of transient global cerebral ischemia^32^,^33^. The BCCAO mimics the systemic decrease in cerebral blood flow, affects the entire brain especially in hippocampus^34^. During the operation procedure, regional cerebral blood flow (rCBF) measurement was taken and only the mice that had <10% residual cortical microperfusion after BCCAO were included for further study. In the present study, we found that ACEA protected hippocampal neurons exposed to OGD/R *in vitro* and improved the neurological scores and eliminated apoptosis induced by BCCAO *in vivo*. Moreover, the cell-permeant CB1 receptor antagonist AM251 reversed the ACEA-induced benefits, while the cell-impermeant CB1 receptor antagonist hemopressin just partially did. These findings suggest that ACEA-induced reduction of neuronal damage may be partially mediated by up-regulating mtCB1R.

Mitochondrial dysfunction is thought to be an inhibition of mitochondrial ATP production, leading to neuronal cell death after cerebral I/R injury^6^,^7^,^35^. Besides the early damage such as mitochondrial transition pore opening, calcium accumulation and decreased glucose utilization^36^, delayed mitochondrial damage may occur within 4–6 h after I/R and affect the activities of mitochondrial complexes, which results in permanent mitochondrial failure^7^,^37^. The mitochondrial electron transport chain (ETC) includes two sites for electron entry. The major one is complex I (NADH dehydrogenase) and the minor one is complex II (succinate dehydrogenase). Electrons offered to either of the two complexes are transferred to complex III (ubiquinol cytochrome c reductase) and subsequently transferred to complex IV (cytochrome c oxidase, CcO) which is the rate-limiting enzyme in electron transfer^38^. In the present study, we chose to detect the activities of three representative complexes, complexes I, II and IV, after OGD/R. The activities of complexes I and IV were significantly decreased at 2 and 24 h after reoxygenation, while the activity of complex II was reduced just at 24 h after reoxygenation; these findings are basically in accordance with a previous study^39^. ACEA ameliorated the reduction of complexes I and IV activities after OGD/R, but had no significant effect on improving complex II activity. MMP establishes a proton gradient across the mitochondrial membrane. During cerebral ischemia, mitochondrial membrane destabilization disturbs the proton gradient and results in the opening of mitochondrial permeablity transition pore (mPTP), releasing mitochondrial components to initiate the apoptotic cascade^40^,^41^. In this study, ACEA improved the mitochondrial membrane potential depolarization after OGD/R. The cell-permeant CB1 receptor antagonist AM251 significantly reversed the ACEA-induced benefits on mitochondrial complexes activities and membrane potential, while the cell-impermeant CB1 receptor antagonist hemopressin did not or just partially reversed the benefits, suggesting that ACEA-induced amelioration of mitochondrial functions may be mediated by up-regulating mtCB1R.

To further clarify the protection of mtCB1R against mitochondrial injury, we employed a Ca^2+^-induced injury model in purified intact mitochondria isolated from normal hippocampal neurons. Ca^2+^-induced mPTP opening has become a widely used model for assessing the effects of drugs on the mPTP^42^. Opening of mPTP can increase mitochondrial inner membrane permeability to solutes with a molecular mass less than 1.5 kDa, causing mitochondrial swelling, which can be evidenced by the reduction in absorbance at 520 nm^43^. In this study, Ca^2+^ induced a noticeable mitochondrial swelling which was followed by a decline of MMP. ACEA-induced mtCB1R activation markedly prevented Ca^2+^-induced mitochondrial swelling and increased the MMP.

However, some limitations of our research should be noted. First, because there are no selected mtCB1R knock-out mouse at present, we just used the regular methods to prove our hypothesis in this study. Second, the usage of cannabis to induce neuroprotection may be limited due to its side effects, including addiction, psychoactivity, tolerance and cytotoxicity^17^,^18^. In the present study, we up-regulated mtCB1R selectively and observed neuroprotection. However, whether the selective up-regulation of mtCB1R brings about fewer side effects still needs further investigation.

In conclusion, this study demonstrated that mtCB1R is involved in ACEA-induced protective effects on neurons and mitochondrial functions, suggesting the mtCB1R may be a potential novel target for the treatment of brain ischemic injury.

## Methods

### Animals and drugs

All animal procedures used in this study were approved by the Ethics Committee for Animal Experimentation of the Fourth Military Medical University and was conducted according to the Guidelines for Animal Experimentation of the Fourth Military Medical University (Xi’an, China). Male C57BL/6 mice (age: 12 weeks; weight: 20–24 g) were provided by the Experimental Animal Center of the Fourth Military Medical University. Mice were kept in cages under the following controlled conditions: a 12-hour light/dark cycle, 21 ± 2 °C, 60–70% humidity, and free access to rodent diet and water.

The selective CB1 receptor agonist, arachidonyl-2-chloroethylamide (ACEA), was dissolved in dimethylsulfoxide (DMSO). The drug was administered intraperitoneally at a dose of 1.5 mg/kg according to a previous study^24^. Hemopressin trifluoroacetate salt, a selective CB1 receptor antagonist, is unable to penetrate plasma membranes^19^,^44^. Therefore, hemopressin (dissolved in H_2_O) can be used as a cell-impermeant CB1 receptor antagonist, which is administered intraperitoneally at a dose of 1 mg/kg for mouse and 10 μM for cells^19^,^45^. All of these agents were obtained from Sigma-Aldrich (St. Louis, USA).

Another selective CB1 receptor antagonist, AM251, which is lipophilic and can penetrate plasma membranes, was dissolved in DMSO before use^19^. AM251 was used at doses of 1 mg/kg for mouse and 10 μM for cells based on previous studies^46^. The agent was obtained from Tocris Bioscience (Bristol, UK). 2-(2-methoxy-4-nitrophenyl)-3-(4-nitrophenyl)-5-(2,4-disulfophenyl)-2H-tetrazolium, monosodium salt (WST-8) was purchased from Sigma-Aldrich (St. Louis, USA). The lactate dehydrogenase (LDH) kit was provided by the Jiancheng Bioengineering Institute (Nanjing, China). The ROS detection reagents were obtained from Invitrogen (Carlsbad, USA).

### Experimental protocols

**Figure 9:**
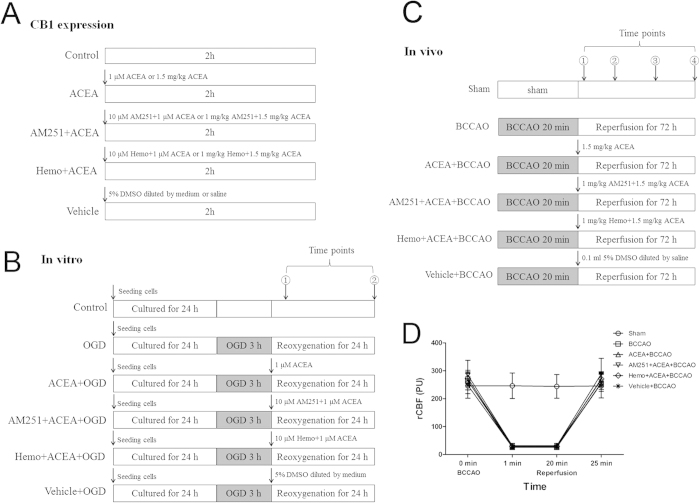
Experimental protocol and regional cerebral blood flow (rCBF). (**A**) Protocol 1: Detection of CB1 receptor expression *in vitro and in vivo*. (**B**) Protocol 2: ACEA treatment *in vitro*. ①: at 2 h after reoxygenation, the neuronal mitochondria were purified for the detection of CB1 receptor expression and mitochondrial function assay; ②: at 24 h after reoxygenation, cell viability was measured and the neuronal mitochondria were purified for mitochondrial function assay. (**C**) Protocol 3: ACEA treatment *in vivo*. ①: at 2 h after reperfusion, mitochondria were isolated for the measurement of CB1 receptor expression; At 24 h (②) and 48 h (③) after reperfusion respectively, neurological tests were conducted; at 72 h (④) after reperfusion, neurological tests were performed, and neuronal apoptosis and apoptosis markers were assessed. (**D**) Changes of rCBF in C57BL/6 mice. The rCBF was reduced to <10% of the pre-ischemic baseline value immediately after ischemia and was maintained for 20 min. The rCBF returned to the pre-ischemic value at 5 min after reperfusion. OGD: oxygen-glucose deprivation; BCCAO: bilateral common carotid artery occlusion; Hemo: hemopressin.

For *in vitro* experiments, to determine the effect of drugs on CB1 receptor expression, five groups of hippocampal neurons (n = 5) were defined as follows: Control, ACEA, AM251 + ACEA, Hemo + ACEA, and Vehicle groups (Fig. 9A). The control cells were cultured in drug-free medium, while cells in other four groups received 1 μM ACEA, 10 μM AM251 + 1 μM ACEA, 10 μM hemopressin + 1 μM ACEA, and vehicle (5% DMSO, diluted by medium), respectively. Mitochondrial proteins were extracted from all groups of cells for Western blot analysis at 2 h after drugs administration. To evaluate the effect of drugs on cell injury and CB1 receptor expression, there were six groups of hippocampal neurons (Fig. 9B). Apart from Control group, other five groups of cells were exposed to OGD for 3 h. The cells in the ACEA + OGD, AM251 + ACEA + OGD, Hemo + ACEA + OGD, and Vehicle + OGD groups received 1 μM ACEA, 10 μM AM251 + 1 μM ACEA, 10 μM hemopressin + 1 μM ACEA, and vehicle (5% DMSO diluted by medium), respectively, at the onset of reoxygenation. Mitochondrial protein was extracted from neurons for Western blot analysis at 2 h after reoxygenation (n = 5). Cell injury was then evaluated in terms of WST-8 cell viability (n = 8), and LDH release (n = 6), apoptotic rate (n = 5) and intracellular ROS level (n = 6) at 24 h after reoxygenation. To detect the mitochondrial function after OGD/R, neuronal mitochondria were purified at 2 and 24 h after reoxygenation respectively (Fig. 9B, n = 6).

For *in vivo* experiments, to detect the expression of mtCB1R at different time points after I/R, fifty-five C57BL/6 mice were randomly divided into three groups: Sham (n = 5), BCCAO (n = 25) and ACEA + BCCAO (n = 25) groups. Mitochondria protein were isolated at 2 h, 6 h, 24 h, 48 h and 72 h after reperfusion respectively and the expression of mtCB1R was assessed by Western blot analysis. To determine the effect of drugs on CB1 receptor expression, forty C57BL/6 mice were randomly divided into five groups (n = 8): Control, ACEA, AM251 + ACEA, Hemo + ACEA and Vehicle groups (Fig. 9A). Mice in the Control group were untreated, the other four groups received 1.5 mg/kg ACEA, 1 mg/kg AM251 + 1.5 mg/kg ACEA, 1 mg/kg hemopressin + 1.5 mg/kg ACEA, and 0.1 ml vehicle (5% DMSO diluted by saline), respectively. The expression of mtCB1R was assessed by Western blot analysis (n = 5) and immunocytochemistry for electron microscopy (n = 3) at 2 h after the administration. To evaluate the effect of drugs on cerebral I/R injury and CB1 receptor expression, one hundred and eight C57BL/6 mice were randomly divided into six groups (n = 18) (Fig. 9C). Except for Sham group, mice from the other five groups were subjected to bilateral common carotid artery occlusion (BCCAO) for 20 min and only the mice whose regional cerebral blood flow (rCBF) remained <10% of pre-ischemic baseline were included (Fig. 9D). The same surgical procedure was performed in Sham group; however, the occlusion was omitted. Mice in the ACEA + BCCAO, AM251 + ACEA + BCCAO, Hemo + ACEA + BCCAO, and Vehicle + BCCAO groups received 1.5 mg/kg ACEA, 1 mg/kg AM251 + 1.5 mg/kg ACEA, 1 mg/kg hemopressin + 1.5 mg/kg ACEA, and 0.1 ml vehicle (5% DMSO diluted by saline), respectively, at the onset of reperfusion. The expression of CB1 receptor was assessed by Western blot analysis (n = 5). Neurological tests were conducted at 24, 48 and 72 h after reperfusion (n = 13). Neuronal apoptosis was assessed by TUNEL staining (n = 5) and apoptosis markers (n = 5) at 72 h after reperfusion. To observe the mitochondrial structure, the mouse CA1 hippocampal neurons were observed by transmission electron microscopy at 72 h after reperfusion (n = 3).

A supplementary file shows the detailed materials and methods concerning cell culture, OGD/R model, cell viability analysis, global cerebral ischemia and reperfusion, neurological scores, TUNEL staining, isolation of mitochondria, Western blot, immunocytochemistry for electron microscopy, semi-quantification of mtCB1R, transmission electron microscopy, mitochondrial enzyme assays and MMP, mPTP detection (see Supplementary Information).

### Statistical analysis

SPSS 13.0 for Windows (SPSS Inc., Chicago, USA) was used to conduct statistical analyses. All values, except for neurological scores, were presented as the mean ± SD, and were analyzed by one-way ANOVA. Between-group differences were detected with post-hoc Student-Newman-Keuls tests. The neurological scores were expressed as the median and were analyzed with Kruskal-Wallis tests followed by the Mann-Whitney U tests with Bonferroni correction. *P* < 0.05 was considered statistically significant.

## Additional Information

**How to cite this article**: Ma, L. *et al.* Mitochondrial CB1 receptor is involved in ACEA-induced protective effects on neurons and mitochondrial functions. *Sci. Rep.* **5**, 12440; doi: 10.1038/srep12440 (2015).

## Supplementary Material

Supplementary Information
