# Supplementary material for: Mitochondrial CB1 receptor is involved in ACEA-induced protective effects on neurons and mitochondrial functions
Source: Sci Rep. 2015 Jul 28;5:12440. doi: 10.1038/srep12440 (PMC4516969; doi:10.1038/srep12440)
Supplement: Supplementary Information [file srep12440-s1.doc]

**Mitochondrial CB1 receptor is involved in ACEA-induced protective effects on neurons and mitochondrial functions**

Lei Ma^1^, Ji Jia^1^, Wen Niu^2^, Tao Jiang^1^, Qian Zhai^1^, Lei Yang^1^, Fuhai Bai^1^, Qiang Wang^1^*, Lize Xiong^1^*

**Detailed materials and methods**

**Cell culture and OGD/R model.** Hippocampal tissues dissected from C57BL/6 mouse embryos at the E18-E19 developmental stage were incubated for 15 min at 37 °C in Ca^2+^ and Mg^2+^-free Hank's balanced salt solution (HBSS) (Invitrogen, San Diego, USA). Tissues were then dissociated and the cells were cultured on poly-L-lysine coated 6-well plates for 4 h at 37 °C. After attachment of the cells to the plate, the medium was replaced with neurobasal medium containing B27, 1 % L-glutamine and 1 % penicillin/streptomycin in a humidified atmosphere containing 5 % CO_2_ and 95 % normal air. The medium was replaced every 2 days. Experiments were performed in 8- to 10-day-old cultures. Then, the cells were characterized by immunohistochemical staining for the expression of neurofilament and fibrillary acidic proteins. Approximately 95 % of the cells in such cultures were shown to be neurons based on this characterization.

To induce OGD, cultured hippocampal neurons were washed three times and incubated with glucose-free Earle’s balanced salt solution at pH 7.4. Then, the cells were incubated in a sealed box containing 95 % N_2_ and 5 % CO_2_ at 37 °C for 3 h. After OGD, the cells were reoxygenated and incubated in normal high-glucose DMEM medium under 5 % CO_2_ and 95 % air at 37 °C for 24 h.

**Assessment of cell viability.** Hippocampal neurons were cultured in 96-well plates at a density of 1 × 10^5^ cells/well. After the treatments described, cell viability was evaluated by the WST-8 dye. WST-8 dye (20 μl) was added into each well and after incubation at 37 °C for 3 h. The absorbance was measured at a wavelength of 450 nm using a spectrophotometer (Tecan, Switzerland).

**Measurement of lactate dehydrogenase (LDH) release.** Hippocampal neurons were cultured in 24-well plates at a density of 2 × 10^5^ cells/well. After treating the cells, supernatants were removed from each well to evaluate LDH levels using the LDH kit according to the manufacturer’s instructions. Briefly, 100 μl of cell-free supernatant, 250 μl of buffer, and 50 μl of coenzyme were mixed homogeneously and the supernatant was incubated with this reaction mixture for 15 min at 37 °C. Then, 250 μl of 2, 4-dinitrophenylhydrazine substrate was added to the mixture and incubated for an additional 15 min at 37 °C in the dark. Finally, 2.5 ml of 400 mM of NaOH was added into the mixture to stop the reaction. After 3 min, the absorbance of the mixture was determined at 440 nm by spectrophotometry. The absorbance of the sample blank, standard, and standard blank was measured at the same time. LDH activity was calculated according to the following formula: LDH activity (U/L) = × 2 × 1000 U/L.

**Flow cytometry analysis for cell apoptosis.** The rate of apoptosis in neurons was evaluated by flow cytometry (BD, USA). Briefly, cells were cultured in a 6-well plate at a density of 5 × 10^5^ cells/well. After treatment, the cells were harvested by centrifugation at 1,000 rpm for 5 min. After two washes with ice-cold phosphate buffered saline (PBS), the cells were resuspended in binding buffer at a density of 1 × 10^6^ cells/ml. Then, 5 µl of fluorescein 5-isothiocyanate [2-(3,6-dihydroxy-9H-xanthen-9-yl)-5-isothiocyanatobenzoic acid] (FITC)-conjugated anti-annexin-V staining antibody and 2 µl of propidium iodide (PI) solution were added to 100 µl of the binding buffer. After thorough mixing, the cells were incubated for 15 min at room temperature in dark, and the apoptotic rate was assessed.

**Measurement of intracellular reactive oxygen species (ROS).** Intracellular ROS was detected by ROS detection reagents utilizing the ability that ROS oxidized nonfluorescent 2,7-dichlorofluorescin diacetate (DCFH-DA) into fluorescent dichlorofluorescein (DCF). DCFH-DA were added to the culture media at a final concentration of 10 μM and incubated at 37 °C for 30 min. Then, the neurons were rinsed twice with PBS and immediately observed under a fluorescence microscope (Leica DMI6000B, Leica Microsystems GmbH, Wetzlar, Germany) with excitation of 488 nm and emission of 525 nm. Images were analyzed using Image Pro-Plus software (IPP 6.0, Media Cybernetics, Silver Spring, USA).

**Global cerebral ischemia and reperfusion.** BCCAO was used as a model of global cerebral ischemia ^1, 2^. Surgical operation was performed by a person who was blinded to the animal groups. Mice were anesthetized with 3 % isoflurane. After induction, the concentration of isoflurane was maintained at 1.5 %, which was supplied by a specially devised facemask. A midline incision was made between the neck and sternum to expose the trachea. Both the right and left common carotid arteries were located according to the sternocleidomastoid and carefully separated from the surrounding tissues and vagus nerve. Cerebral ischemia was induced by clamping both the arteries with two miniature artery clips. The laser Doppler flowmeter (PeriFlux System 5000; Perimed, Stockholm, Sweden) was used to measure regional cerebral blood flow (rCBF) (2-3 mm lateral to the bregma) from the time of anesthetic induction to 5 minutes after reperfusion ^1, 3^. In our experimental model, only mice with a mean cortical CBF reduced to < 10 % of the pre-ischemic value were used for data analysis (Fig. 9D). Totally, 19 mice were excluded for not meeting rCBF inclusion criteria and 23 mice were excluded for surgical failure. After 20 minutes of cerebral ischemia, the clips were removed from both arteries to allow reperfusion of the carotid arteries. The incision was sutured using 4-0 Mersilk (Ethicon, Johnson & Johnson, Somerville, USA). During the surgical procedure, the pericranial temperature was monitored using a temperature probe and maintained at 37.0 °C to 37.5 °C with a heating pad. After surgery, animals were placed in warm surroundings (30-33 °C) to avoid results bias due to hypothermia.

**Neurological scores.** The treated mice were allowed to recover for 24 h before subsequent tests. Neurological scores was performed by a person who was blinded to the animal groups Mice were subjected to a modified neurologic examination designed to detect motor deficits. Briefly, mice were placed on a 10- to 20-cm screen (grid size 0.2 × 0.2 cm) that could be rotated from 0° (horizontal) to 90° (vertical). The mouse was placed on the horizontal screen, and the screen was then rotated into the vertical plane. The duration that the mouse was able to hold on to the vertical screen was recorded up to a maximum of 15 s (corresponding to a maximum of 3 points). Next, the mouse was placed at the center of a horizontal wooden rod (1.5 cm in diameter), and the duration that the mouse was able to remain balanced on the rod was recorded up to a maximum of 30 s (corresponding to a maximum of 3 points). Finally, a prehensile traction test was administered. The time that the mouse was able to cling to a horizontal rope was recorded for a maximum of 5 s. From these tests, a total motor score (TMS) (9 possible points) was calculated. The neurologic tests were determined at 24-, 48-, and 72-h post-reperfusion by an observer who was unaware of the grouping. The TMS system used in the current study has been shown to be an accurate method for the evaluation of global cerebral ischemia in mice ^2, 4^.

**TUNEL staining.** For detection of in situ DNA fragmentation, terminal deoxynucleotidyl transferase-mediated dUTP-biotin nick end labeling (TUNEL) staining was performed using an In Situ Cell Death Detection Kit (Roche Diagnostics, Mannheim, Germany) according to the manufacturer’s instructions as described in our previous study ^5^. The total number of TUNEL-positive neurons in the CA1 region were counted in three different fields for each section in a blind manner by fluorescence microscopy at ×400 magnification (BX-60; Olympus, Tokyo, Japan). Data from five animals at each stage were averaged.

**Isolation of mitochondria.** Mitochondria were isolated using a Qproteome mitochondria isolation kit (Qiagen, Hilden, Germany) according to the manufacturer's specifications. A small quantity of purified mitochondria was observed by electron microscopy (Fig. 8B). The mitochondria were round or oval with the morphological integrity of membrane and cristae structures, indicating that the used method of isolating mitochondria was reliable.

**Western blot analysis.** Hippocampus samples were used for mitochondrial isolation as previously described. The total protein concentration was determined by the Bradford method, and Western blot analysis was performed as previously described ^6^. The following primary antibodies were used: anti-CB1 receptor polyclonal antibody (1:200, Abcam, Cambridge, UK), anti-COX IV polyclonal antibody (1:1,000, Abcam, Cambridge, UK), anti-GAPDH polyclonal antibody (1:1,000, CWBIO, Beijing, China), anti-β-actin (1:5000, Sigma-Aldrich, St. Louis, USA), anti-cleaved caspase-3 (1:800, Cell Signaling, USA). Appropriate secondary horseradish peroxidase-conjugated goat anti-rabbit or goat anti-mouse antibodies (1:10,000, CWBIO, Beijing, China) were used. Antigens were detected using the chemiluminescence technique (Amersham Pharmacia Biotech Piscataway, USA). Image analysis was accomplished with the assistance of computerized analysis software (Bio-Rad Laboratories, Hercules, USA).

**Immunocytochemistry for electron microscopy.** Mice hippocampal CA1 regions were processed for electron microscope pre-embedding immunogold labeling as previously described ^6^. A CB1 receptor polyclonal antibody (1:200, Abcam, Cambridge, UK) was used for immunocytochemical staining of 50-μm-thick hippocampal sections.

**Semi-quantification of mtCB1.** The detailed process has been described previously ^6, 7^. Briefly, mtCB1 labeling density was calculated on the area of mitochondria. To avoid false negatives, only the first 1.5 μm from the surface of the tissue block were cut for 80 nm ultrathin sections and the same position and depth were selected.

**Transmission electron microscopy.** Mice were deeply anesthetized and transcardially perfused with normal saline and fixative composed of 4 % paraformaldehyde and 2.5 % glutaraldehyde. The hippocampal CA1 regions were removed for postfix with 3 % glutaraldehyde (pH 7.4, 4°C) and then fixed again in 1 % osmium tetroxide, dehydrated in graded ethanol, embedded in epon. 50-μm-thick hippocampal sections were cut with amicrotome (Bromma, Sweden), counterstained with uranyl acetate and lead citrate, and observed with a JEM-2000EX transmission electron microscope (JEOL, Japan).

**Determination of respiratory chain complexes activities and mitochondrial membrane potential.** Activities of complex I (NADH-ubiquinone oxidoreductase), complex II (succinate dehydrogenase), and complex IV (cytochrome c oxidase) of the mitochondrial electron transport chain were measured spectrophotometrically at 30 °C using the previously described methods ^8^.

Mitochondrial membrane potential (MMP) was determined using JC-1 (Sigma-Aldrich, St. Louis, USA). Mitochondria samples (0.5 mg/ml, 1 ml) were incubated with 19 ml JC-1 staining buffer according to the manufacturer’s instruction. At the end of the experiments, valinomycin was added as a negative control. Fluorescence intensity was determined at 37 °C in a fluorescence spectrophotometer (Tecan, Switzerland). The ratio of aggregates (red, 590 nm) to monomer (green, 525 nm) was calculated as an indicator of MMP.

**Measurement of mitochondrial permeability transition pore opening.** mPTP opening was assessed by Ca^2+^-induced mitochondrial swelling as previously described ^9^. Briefly, 1 ml of respiration buffer plus 10 mM succinate was used to suspend 1 mg of isolated mitochondria. After 5 min of pre-incubation at 36 °C and baseline measurement, ACEA and Ca^2+^ were added respectively. Ca^2+^-induced mitochondrial swelling was measured by the loss of absorbance at 520 nm using a spectrometer.

**References**

1. Kitagawa, K. *et al*. Cerebral ischemia after bilateral carotid artery occlusion and intraluminal suture occlusion in mice: evaluation of the patency of the posterior communicating artery. *J Cereb Blood Flow Metab* **18**, 570-579 (1998).
2. Homi, H.M. *et al*. Severe hypotension is not essential for isoflurane neuroprotection against forebrain ischemia in mice. *Anesthesiology* **99**, 1145-1151 (2003).
3. Wacker, B.K., Park, T.S. & Gidday, J.M. Hypoxic preconditioning-induced cerebral ischemic tolerance: role of microvascular sphingosine kinase 2. *Stroke* **40**, 3342-3348 (2009).
4. Dong, H. *et al*. Preconditioning with hyperbaric oxygen and hyperoxia induces tolerance against spinal cord ischemia in rabbits. *Anesthesiology* **96**, 907-912 (2002).
5. Wang, Q. *et al*. Pretreatment with electroacupuncture induces rapid tolerance to focal cerebral ischemia through regulation of endocannabinoid system. *Stroke* **40**, 2157-2164 (2009).
6. Bénard, G. *et al*. Mitochondrial CB₁ receptors regulate neuronal energy metabolism. *Nat Neurosci* **15**, 558-564 (2012).
7. Puente, N. *et al.* Polymodal activation of the endocannabinoid system in the extended amygdala. *Nat Neurosci* **14**, 1542-1547 (2011).
8. Benard, G. *et al*. Physiological diversity of mitochondrial oxidative phosphorylation. *Am J Physiol Cell Physiol* **291**, C1172-C1182 (2006).
9. Sullivan, P.G., Thompson, M.B. & Scheff, S.W. Cyclosporin A attenuates acute mitochondrial dysfunction following traumatic brain injury. *Exp Neurol* **160**, 226-234 (1999).
